# Rigorous intensity and phase-shift manipulation in optical frequency conversion

**DOI:** 10.1038/srep27457

**Published:** 2016-06-07

**Authors:** Bo Yang, Yang-Yang Yue, Rong-er Lu, Xu-Hao Hong, Chao Zhang, Yi-Qiang Qin, Yong-Yuan Zhu

**Affiliations:** 1 National Laboratory of Solid State Microstructures and Collaborative Innovation Center of Advanced Microstructures and Key Laboratory of Modern Acoustics, Nanjing University, Nanjing 210093, China; 2 College of Engineering and Applied Sciences, Nanjing University, Nanjing 210093, China; 3 School of Physics, Nanjing University, Nanjing 210093, China

## Abstract

A simple method is employed to investigate the nonlinear frequency conversion in optical superlattices (OSL) with pump depletion. Four rigorous phase-matching conditions for different purposes are obtained directly from the nonlinear coupled equations, and the resulting OSL domain structures are generally aperiodic rather than periodic. With this method, not only the intensity but also the phase-shift of the harmonic waves can be manipulated at will. The second-harmonic generation of Gaussian beam is further investigated. This work may provide a guidance for the practical applications of designing nonlinear optical devices with high conversion efficiency.

The phase-matching (including quasi-phase-matching (QPM) proposed by J. A. Armstrong *et al.* in^1^) is of fundamental importance in nonlinear optics. The QPM can be realized in the nonlinear material with nonlinear susceptibility χ^(2)^ being artificially modulated^2^ (the so-called optical superlattice (OSL) or nonlinear photonic crystal). Thanks to the improvement in poling technique, the QPM has been widely used nowadays in the frequency conversion with one-dimensional (1D) or two-dimensional (2D) OSLs^3^,^4^,^5^,^6^. In ultrafast optics QPM can be used for pulse compression and multiple parametric generations^7^,^8^,^9^,^10^,^11^. In quantum optics, entangled photons can be produced efficiently in the spontaneous parametric frequency conversion process with QPM method^12^,^13^,^14^,^15^,^16^.

It is known that the traditional QPM condition is deduced under the pump undepleted approximation (small signal approximation). Practically, the energy transfer between the fundamental wave (FW) and the harmonic wave should be considered because of the FW depletion. Several methods have been investigated to study this problem. In^17^, K. C. Rustagi *et al.* provided a rigorous analytical solution in the form of elliptic function to analyze the second-harmonic generation (SHG) and three-wave mixing^17^, where the ideal QPM stack and the tolerance of the domain length was discussed. They pointed out that the structure can be tailored according to the initial amplitude and the relative phase-shift in each stack. The result was further analyzed and numerically verified^18^,^19^. In these studies, the main attention was focused on the perfect phase matching for high conversion efficiency. Few attention has been drawn to the phase-shift manipulation. In this paper, we employed an intuitive and simple method to study the nonlinear optical interactions with pump depletion. The manipulation of the energy transfer and phase-shift during the nonlinear process were analyzed and four different rigorous conditions (including the perfect phase matching) were derived for various purposes. And, we applied this method to the SHG of 2D Gaussian beams considering the pump depletion.

## Intensity manipulation theory

**Figure 1:**
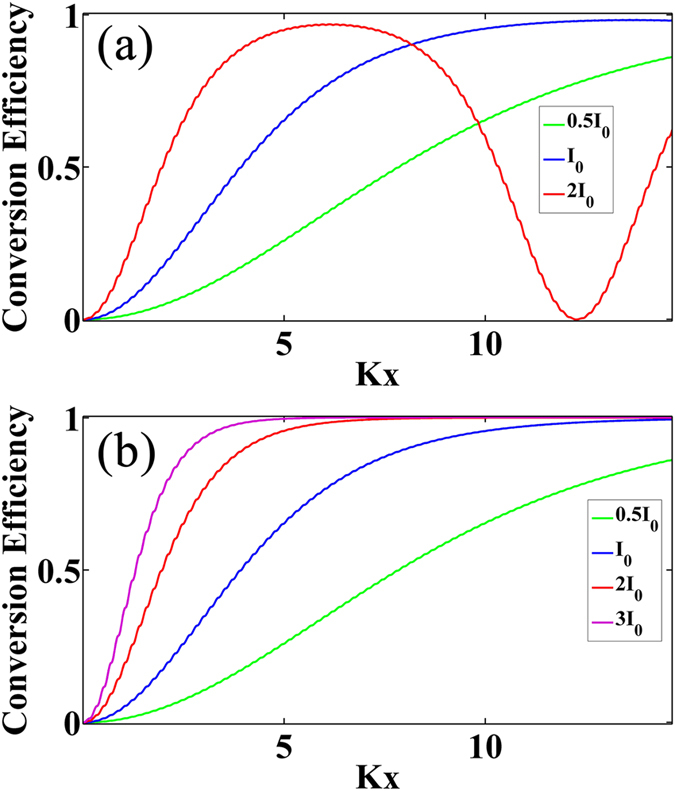
Conversion efficiency of SHG with different incident FW intensities under pump depletion. (**a**) the conventional QPM configuration, the crystal is periodical. I_0_ represents the intensity of FW leading to the optimal output. (**b**) the rigorous phase matching configuration, the crystals are determined by Eq. (4).

Considering a SHG process in the OSL, the behavior of the harmonics is governed by the coupled wave equations^20^:


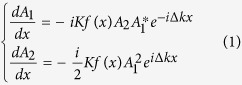


where *K* is the coupling constant, *f* (*x*) is the OSL structure function, *A*_1_ and *A*_2_ is the amplitude for the FW and SHW, respectively. Usually *f* (*x*) is a real function with binary values (1 for positive domains and −1 for negative domains), and it contains multiple reciprocals in its Fourier spectrum. For the QPM mode, the OSL is periodical and the structure function can be easily set to be *f* (*x*) = sign{cos (Δ*kx*)}. In a periodic OSL, if the crystal length is fixed, the SHW is decided by the coupling constant and the initial state of the FW as shown in Fig. 1(a). Clearly the optimal output of the SHG can be obtained by the FW with the initial intensity being I_0_ and the initial phase being 0. Here the x-coordinate (the length of OSL) is normalized by the coupling constant. It can be seen that if the FW intensity is varied, the conversion efficiency will decrease accordingly. It means that the conventional QPM is not rigorous under the pump depletion case. Therefore, to find out a suitable OSL for the optimal output for the incident FW is a critical topic.

Different from the analysis using elliptic function solution in K. C. Rustagi *et al.*’s work^17^, here we employ a more simple method for the design of the OSL structure considering the pump depletion. The basic idea is rather simple, that is, to ensure the monotonous increasing of the SHW in a SHG process, the following rule should be held in the whole SHG process,


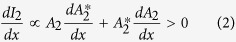


The detailed expressions for 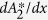 and 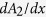 can be obtained from Eq. (1), substituting them into Eq. (2) we get


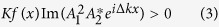


where Im(z) represents the imaginary part of *z*. Since *K*, *f*(*x*) and 
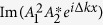
 are all real and *K* > 0, the only condition for *f* (*x*) to fulfill Eq. (3) is immediately determined by:


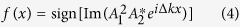


where


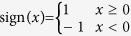


Equation (4) is what we need. When this condition is satisfied, the intensity of the SHW will increase monotonously with the sample length. A typical result for the SHG process under this condition is shown in Fig. 1(b). Governed by the OSL decided by Eq. (4), no matter how the intensity of the incident FW changes, the OSL always ensures an optimal output. Therefore we call it the Type-1 rigorous phase matching (RQPM) condition. In this situation all the energy is finally transferred to the SHW and never fall back. In general, the OSL structure obtained from Eq. (4) is aperiodic and depends on the input intensity of the FW. This is different from the conventional QPM, which is independent of the FW intensity and results in a period structure. It can be proven that the Type-1 RQPM condition is equivalent to the result from the work by K. C. Rustagi *et al*^17^. (The details can be found in the Supplementary Info.) Different from the consideration of the relative domain length of each stack, we give out a simple expression for the OSL structure. When the conversion efficiency is low, this RQPM structure can degenerate to the conventional periodic structure under the small signal approximation.

With the above idea the energy flow of the nonlinear process can be manipulated precisely. To ensure monotonous decreasing of the SHW, the simple rule which should be used is *dI*_2_/*dx* ≤ 0, repeating the procedure of deducing Eq. (4) we get:


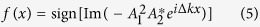


We call Eq. (5) the Type-2 RQPM condition. When this condition is satisfied, the intensity of the SHW will decrease monotonously with the sample length, which is just the opposite of Type-1 RQPM condition. That is to say, under this condition the OSL acts as a “lossy medium” for the SHW, while under Type-1 RQPM condition it acts as a “gain medium”. Note that Eq. (5) has a similar form as Eq. (4) except for a negative sign, but the OSL structure thus obtained is not just a simple reversal of Eq. (4) since the values of *A*_1_ and *A*_2_ are different in general when calculating *f* (*x*) with these two equations.

**Figure 2:**
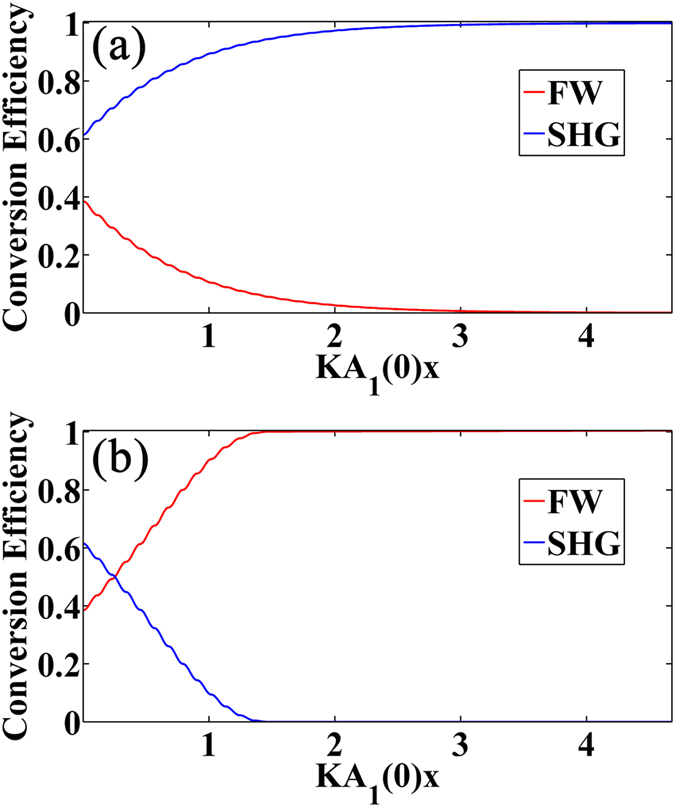
Manipulating the energy flow with the RQPM method. (**a**) the result of Type-1 RQPM condition; (**b**) the result of Type-2 RQPM condition.

To demonstrate the ability of manipulating energy flow, here we consider a SHG process where both *A*_1_ and *A*_2_ have non-zero initial values at *x* = 0, and the ratio of input intensities of two waves is chosen to be about 5:4. Figure 2(a,b) show the numerical results under Type-1 and Type-2 RQPM condition respectively. We can see that the behaviors of these two RQPM conditions are just the opposite. In Fig. 2(a) all the energy transfers to the SHW while in 2(b) all the energy transfers to the FW. Manipulating the energy flow is especially useful in the parametric down conversion process such as difference-frequency generation or optical parametric amplifier.

### Phase-shift manipulation theory and cascaded processes

Nonlinear phase-shifting is another important topic in nonlinear optical processes besides frequency conversion^21^. The RQPM method can be extended to realize monotonous phase-shifting as well. Assuming *y*_*i*_ = |*A*_*i*_| and *ϕ*_*i*_ = arg(*A*_*i*_) (*i* = 1, 2), where 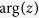 is the complex argument function, substituting them into Eq. (1) and we get


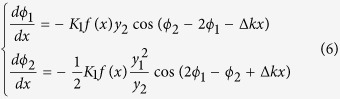


From Eq. (6) we can deduce the RQPM condition of phase-shifting easily. If we apply the rule *dϕ*_2_/*dx* ≥ 0 to the SHG process, we can get


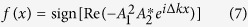


We call it the Type-3 RQPM condition. Under this condition the phase-shift of the SHW will increase monotonously. Similarly, with the rule *dϕ*_2_/*dx* ≤ 0 we can get the Type-4 RQPM condition:


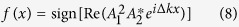


Under this condition the phase-shift of the SHW will decrease monotonously.

**Figure 3:**
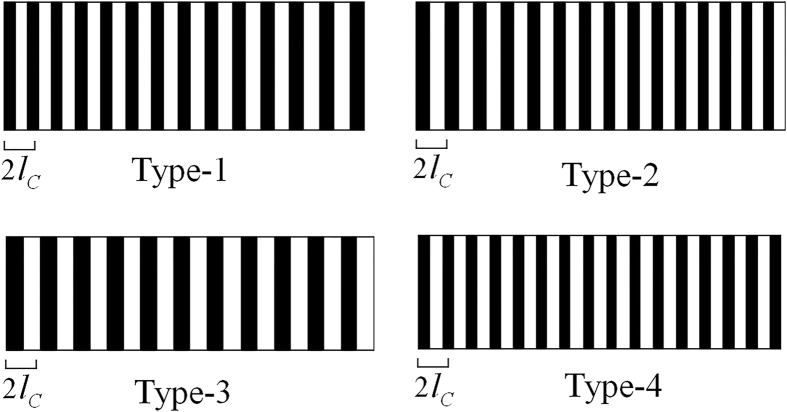
Schematic diagram for the OSL structures under RQPM conditions. *l*_*C*_ is the coherent length which is defined by *l*_*C*_ = (*π*)/(Δ*k*).

Figure 3 shows the numerical results for the OSL structures corresponding to the above 4 RQPM conditions. Type-1 and Type-2 conditions usually result in aperiodic structures. The domain sizes of these two structures are slightly chirped with positive and negative chirp rates respectively. Type-3 and Type-4 conditions usually result in periodic structures. The period of Type-3 structure is always larger than the period of QPM, while the period of Type-4 structure is smaller. And the OSL structures of the four types RQPM conditions are all affected by the initial amplitude and phase of the FW and SHW.

**Figure 4:**
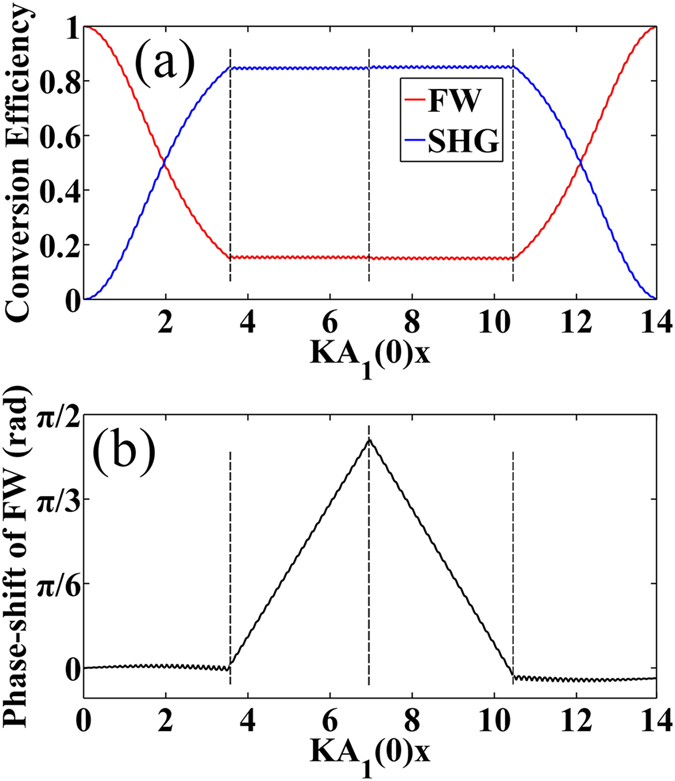
Manipulating both of energy flow and phase-shifting in a multi-region RQPM structure. (**a,b**) shows the intensity distribution and the phase distribution of the harmonic waves under the same initial conditions, respectively.

These RQPM conditions might be useful for designing integrated optical devices. Here we show an example where all of the above 4 types of RQPM conditions are employed in a SHG process. The OSL is divided into four regions, and the structure in the *i*-th region (*i* = 1, 2, 3, 4) is designed to satisfy the Type-*j* RQPM condition (*j* = 1, 3, 4, 2), respectively. Thus the generated SHW is expected to experience intensity increasing, positive phase-shifting, negative phase-shifting and intensity decreasing, respectively. The numerical results for the intensity and phase distribution of the SHW are shown in Fig. 4(a,b). The two adjacent regions are separated by a dashed line, and the initial condition is the same as that used in Fig. 1. We can see that the result coincides well with the expectation.

### Rigorous phase-matching for 2D Gaussian beams

**Figure 5:**
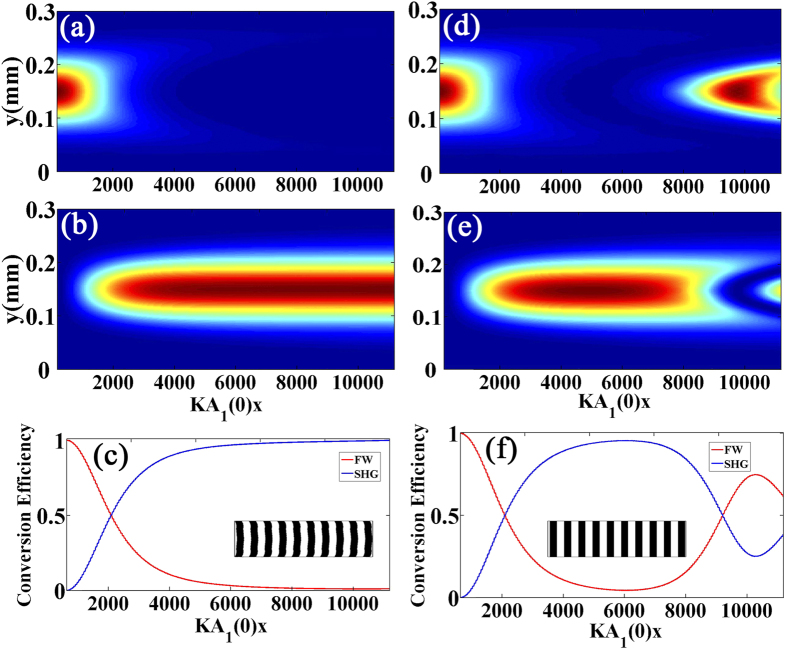
Numerical simulation for the SHG processes involving Gaussian beams. (**a–c**) shows the field distributions of the FW, SHW, and the related conversion efficiency under Type-1 RQPM condition, respectively. (**d–f**) shows the corresponding results in a periodic OSL under conventional QPM condition for comparison. The corresponding OSL structures are shown in the insets of (**c,f**).

The study on wave-front shaping or special beam generation in nonlinear processes has become a hot topic recently, where harmonic waves with complicated wave-front are involved^22^,^23^,^24^,^25^,^26^,^27^. We found that the RQPM method can be applied in this field although it is originated from the 1D coupled wave equations. Numerical simulations have been performed to analyze the SHG processes involving 2D Gaussian beams under Type-1 RQPM condition and conventional QPM condition. The initial wave-front of the FW is a Gaussian beam with a waist radius of ~80 *μm*, and the wavelength of the FW is 1.064 *μm*, and the confocal length is larger than the crystal length. To obtain an efficient SHG, the required OSL structure for RQPM can be decided by 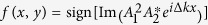, which has the same form as Eq. (4) except that here *A*_1_ and *A*_2_ are functions of two variables, thus it usually results in a 2D OSL structure. It should be noted that Gouy phase shift is automatically taken into account in our method. The simulation results for the FW and SHW in this structure are shown in Fig. 5(a,b), and the conversion efficiency is shown in Fig. 5(c). The corresponding results in a periodic structure with conventional QPM are shown in Fig. 5(d–f) for comparison. It can be observed that the character of monotonously energy transferring for RQPM is kept. Thus higher conversion efficiency for the SHW can be achieved in this process.

In general, the nonlinear frequency conversion is significantly related to the incident FW. That is, the higher incident FW intensity results in a quicker frequency conversion. For the FW with Gaussian intensity profile, the progress of the frequency conversion in a traditional QPM OSL on the transverse section is not uniform, as shown in Fig. 5(d), resulting in an imperfect phase matching. To realize the perfect phase matching with such a non-uniform FW is beyond the ability of traditional QPM method, but might be possible in the RQPM scheme.

## Conclusion

In conclusion, we employed a simple method for designing QPM domain structures, the structure function of the OSL can be deduced directly from the coupled equations. Up to four types of phase matching conditions have been obtained. These conditions are rigorous in theory and can be used for different purposes. The method enables us to manipulate the energy flow and the phase-shift freely during the nonlinear process. It is universal and can be applied to nearly all kind of second-order nonlinear optical processes, and is also possible to be employed to the QPM processes involving special beams.

## Methods

### Derivation of the intensity variation of SHW

In a SHG process, the intensity of the harmonic wave can be written as^20^,


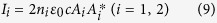


where *I*_*i*_, *A*_*i*_, *n*_*i*_, *ε*_0_, *c* represents the intensity, amplitude, refractive index, permittivity, and light velocity, respectively. The spatial variation of the intensity of SHW can be expressed as,


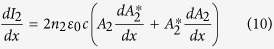


According to the nonlinear coupled equation Eq. (1), we have


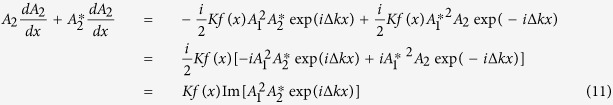


Thus the intensity variation of SHW is decided by


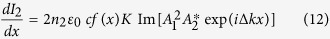


### Configuration for the phase-matching of Gaussian beams

The OSL structure for phase-matching of 2D Gaussian beams is decided by


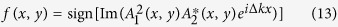


*A*_1_ and *A*_2_ are the local amplitudes of the FW and SHW. To determine *f* (*x*, *y*) we should know the values of *A*_1_ and *A*_2_ at (*x*, *y*), which can be calculated in real time with a finite difference method^28^.

## Additional Information

**How to cite this article**: Yang, B. *et al.* Rigorous intensity and phase-shift manipulation in optical frequency conversion. *Sci. Rep.* **6**, 27457; doi: 10.1038/srep27457 (2016).

## Supplementary Material

Supplementary Information
