# Supplementary material for: Rigorous intensity and phase-shift manipulation in optical frequency conversion
Source: Sci Rep. 2016 Jun 7;6:27457. doi: 10.1038/srep27457 (PMC4895210; doi:10.1038/srep27457)
Supplement: Supplementary Information [file srep27457-s1.doc]

**Rigorous intensity and phase-shift manipulation in optical frequency conversion**

Bo Yang,^1,2^ Yang-Yang Yue,^1,2^ Rong-er Lu,^1,2^ Xu-Hao Hong,^1,3^ Chao Zhang,^1,2,*^ Yi-Qiang Qin,^1,2,†^ and Yong-Yuan Zhu^1,3^

**A comparison between Type-1 RQPM and the results in Ref** [**^1^**](#_ENREF_1)

In 1982, K. C. Rustagi *et al*. provided an analytical solution in the form of elliptic function to analyze the second-harmonic generation (SHG) and three-wave mixing in the Quasi-phase-matching (QPM) configuration with FW depletion [^1^](#_ENREF_1), where the ideal QPM stack was obtained and the tolerance of the domain length was analyzed. They pointed out that the ideal crystals must be tailored according to the amplitude and phase of the initial FW and second-harmonic wave, and the most efficient stack should be determined by the relative phase $\pi$ in each stack, which was further analyzed and numerically verified .

According to Ref [1], the relative phase is defined as,

where , arg is a complex argument function.

Here we make a comparison between Rustagi's theory and our theory. An ideal QPM stack for SHG is determined using Rustagi's method, with the initial parameters of $A_{10}=0.5,\text{ }A_{20}=0$, and a random section of the stack is shown in Figure. S1(a). Using the same initial condition and Type-1 RQPM condition,

$f(x)=\mathrm{sign}[\mathrm{Im}(A_{1}^{2}A_{2}^{*}e^{i\Delta kx})]$,

the required optical superlattice (OSL) structure can be obtained, as shown in Figure. S1(b). It is found that the parameters of the structure is identical with that of Figure. S1(a). Furthermore, the corresponding relative phase in the RQPM configuration is also calculated, as shown in Figure. S1(c). It can be seen from Figure. S1(b) and Figure. S1(c) that the polarization direction of the domain is changed only when the relative phase changes π in a same domain stack, which also agreed well with the result in Ref [1].


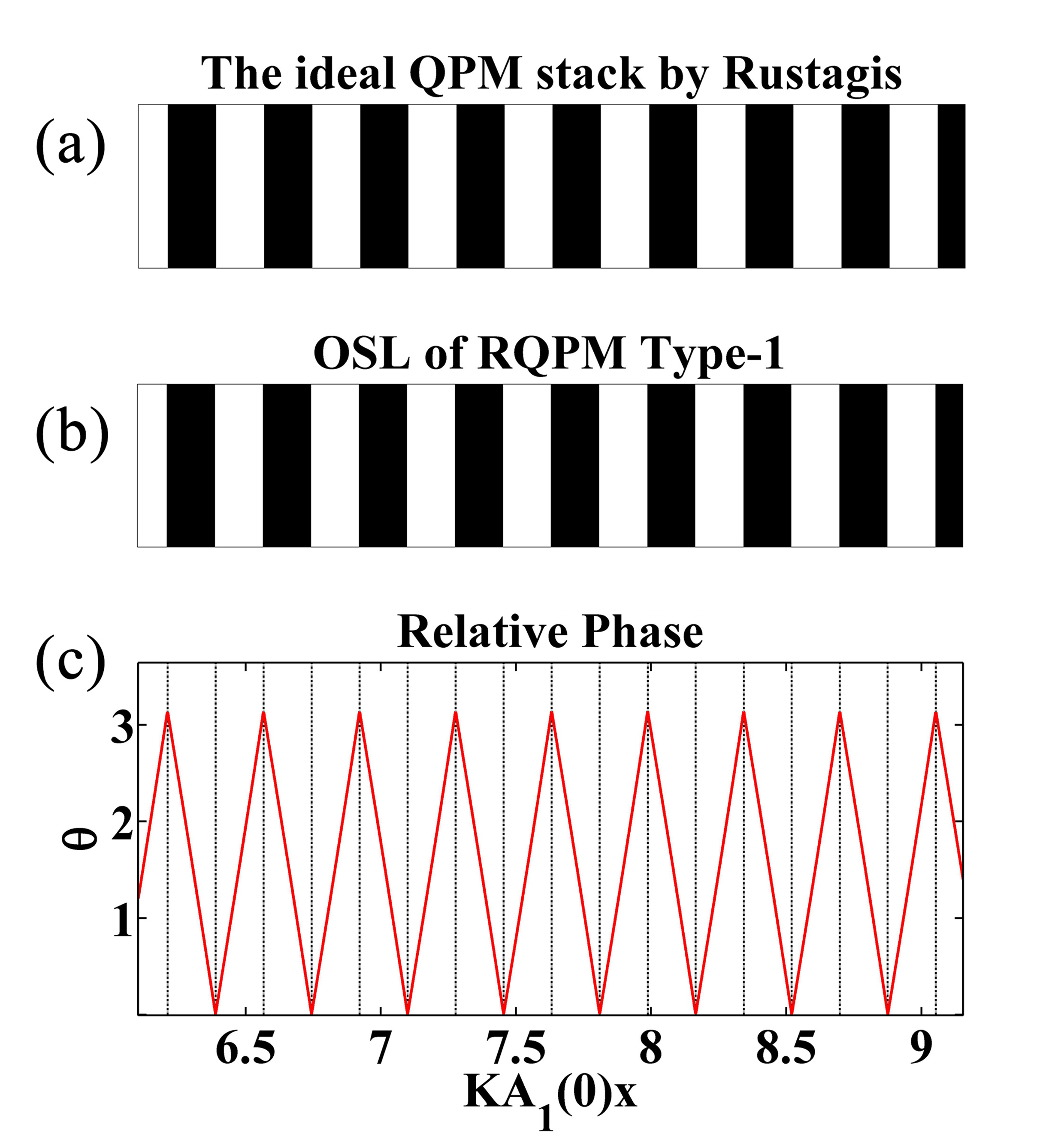


Figure. S1 (a) The ideal QPM stack structure obtained with the method in [1]. (b) OSL structure obtained with Type-1 RQPM. (c) Relative phase in the RQPM configuration. The shown part is chose randomly from the structure. The x coordinates is normalized and the initial condition is set to be $A_{10}=0.5,\text{ }A_{20}=0$.

In summary, the RQPM method is consistence with the method in Ref [1], and both two methods can be used to design the ideal QPM structure. Furthermore, the RQPM theory can be also employed to analyze sum-frequency generation, third-harmonic generation and other nonlinear optical interactions.

1. Rustagi, K. C., Mehendale, S. C. & Meenakshi, S. Optical Frequency-Conversion in Quasi-Phase-Matched Stacks of Non-Linear Crystals. *IEEE J Quantum Elect* **18**, 1029-1041 (1982).

2. Zhao, L. M., Yue, G. K. & Zhou, Y. S. Effect of the Pump Depletion Itself on the Quasi-Phase-Matching for Second-Harmonic Generation. *Epl-Europhys Lett* **99** (2012).

3. Zhao, L. M., Yue, G. K., Zhou, Y. S. & Wang, F. H. Effect of Pump Depletion on Second Harmonic Generation in Multiple Quasi-Phase-Matching Gratings. *Opt Express* **21**, 17592-17601 (2013).
